# Ants as vectors of pathogenic microorganisms in a hospital in São Paulo county, Brazil

**DOI:** 10.1186/1756-0500-7-554

**Published:** 2014-08-20

**Authors:** Heros J Máximo, Henrique L Felizatti, Marcela Ceccato, Priscila Cintra-Socolowski, Ana Laura R Zeni Beretta

**Affiliations:** Postgraduate Program – Master’s Degree in Biomedical Science/Uniararas, Araras, São Paulo Brasil; Health and Agrarian Science Department of Anhanguera University Centre, Leme, São Paulo Brazil; Statistics Department of Federal University of Goiás, Goiás, Brazil; Social Insects Study Centre (CEIS) of Bioscience Institute of Rio Claro, UNESP, Rio Claro, São Paulo Brazil; Postgraduate Program in Biomedical Science/Health Science Nucleus, Hermínio Ometto University Centre (UNIARARAS), Araras, São Paulo Brazil

**Keywords:** Nosocomial infection, Bacteria, Ants, Pathogenic microorganisms, Nosocomial environment

## Abstract

**Background:**

The present study aimed to identify and characterize the presence of bacteria carried by ants, and check the distribution of these ants in the physical confines of a medium-sized hospital in São Paulo county, Brazil.

**Methods:**

The ants were collected from March 2012 to February 2013. Attractive non-toxic baits were used to catch the ants, and the sectors considered for the study were medical wards, outdoor areas, obstetric unit, reception area, kitchen, surgical centres, paediatric clinic and intensive care unit. Captured ants were classified using taxonomic keys and subsequently immersed in Brain Heart Infusion broth.

**Results:**

*Paratrechina* spp. and *Monomorium floricola* ants were found most frequently in the hospital. Ants had a high capacity for carrying bacteria, and the isolates comprised 68.8% Gram-positive, spore-producing bacilli (*Bacillus* spp. and *Listeria* spp.); 14.7% Gram-negative bacilli (*Pseudomonas aeruginosa* and *Klebsiella* spp.); and 16.4% Gram-positive cocci (*Streptococcus* spp. and *Staphylococcus aureus*). Among the areas being evaluated, the medical wards had the largest number of ants captured, and therefore the most bacteria.

**Conclusions:**

Ants in hospitals may carry both Gram-positive and Gram-negative bacteria, and methods of controlling urban ants should be adopted and strictly adhered to, to minimize the risk of infection in hospital patients.

## Background

Ants are social insects that live in symbiosis with humans and readily adapt to urban environments. They can affect the quality of human life, because of the possibility of causing damage and threats to health. The hospital environment is one of the main areas for ants and their presence can facilitate the propagation and spread of pathogenic microorganisms [1, 2].

In Brazil, there are approximately 2,000 known species of ants, and 20–30 are considered to be an urban plague [3]. The dispersion and increasing populations of urban ants are facilitated by several factors, the most important of which are: polygyny, unicolonial populations, migration of colonies, polidomic colonies, reproduction by fragmentation, small nest structure and workers with reduced size and without nuptial flight [3].

The occurrence of ants in hospitals has become a research focus owing to the exposure of patients and health professionals to the risks associated with these insects. The quality of healthcare assistance in urban hospitals also suffers from the problem of an increase in microbial vectors. Studies in two hospitals in Northeast Brazil have warned about the specific role of ants (Hymenoptera: Formicidae) in the transport of pathogenic bacteria associated with hospital environments [4]. Among the factors that favour the presence of ants in urban hospitals are: the arrangement of architectural structures; nearby homes where ants are present, which favours migration of ants; the packaging of some medications may harbour nests of ants, bringing them into the internal environment; the provision and maintenance of air conditioning; the large number of people with clothes and objects that may contain ant nests, and attractions such as food scraps and organic material [5, 6].

Nosocomial infection has attracted much interest in the scientific community because of the high rates of morbidity and mortality in hospitalized patients [7, 8]. Its occurrence depends on the sanitary conditions and the presence of vectors of pathogenic microorganisms. Among social insects, ants make numerous parasitic and mutualistic relationships and develop multiple interactions with animals, plants, fungi and bacteria [2]. This carries a great risk of infection in hospitals on account of the mobility of ants within the hospital environment [7, 9, 10].

Considering the ability of ants to carry and disseminate pathogens in the hospital environment, we aimed to identify and characterize the presence of bacteria associated with ants, and the distribution of these ants within the physical confines of a medium-sized hospital in São Paulo county, Brazil.

## Methods

### Bioethics committee permission

This study was approved under the legal agreement 013/2012 by the Ethics Committee for Animal Use – CEUA of Hermínio Ometto Foundation – UNIARARAS, Araras, São Paulo, Brazil.

### Capture and identification of ants

To catch the ants, we used 15-mL Falcon-type tubes, containing 5 mL sterile non-toxic attractive bait [11], packed in a plastic box (28 × 15 × 13 cm), previously sterilized by non-ionizing radiation. These tubes allowed entry of the ants, and were divided into sampling points in the morning for a period of 2 h (between 10:00 and 12:00 h). The samples were collected monthly during March 2012 to February 2013 in a hospital in São Paulo county, Brazil. The collection points were medical wards, outdoor areas, obstetric unit, reception area, kitchen, surgical centres, paediatric clinic and intensive care unit (ICU). These areas were selected because of the higher incidence of ants and criteria for risk of infection of patients [9, 12]. Captured ants were subjected to morpho-dyeing; cultural, biochemical and microbiological analysis; and subsequently fixed in 80% alcohol. The ants were mounted on entomological pins and identified using pictorial keys [4] keys and complete keys [13]. The classification of ants was performed in collaboration with the Centre for the Study of Social Insects (CEIS) of the Institute of Biosciences of Rio Claro – UNESP, São Paulo, Brazil.

### Microbiological analysis

Captured ants were immersed in Brain Heart Infusion (BHI) broth, incubated in a microbiological growth medium for 24 h at 36 ± 1°C, and samples that showed growth were plated on BHI agar by the method of exhaustion. The isolated pure colonies were seeded in specific environments for isolating particular microorganisms and subjected to morphological, cultural and biochemical analyses [14]. Chromogenic culture media for isolation and identification of Gram-positive cocci, and identification panels for glucose-fermenting and glucose-non-fermenting Gram-negative bacilli (Probac Brasil, São Paulo, Brazil). Microbiological analysis was performed at the Microbiology Laboratory, Anhanguera University Centre, Leme, São Paulo, Brazil and the Centre for Health Sciences, Hermínio Ometto University Centre (UNIARARAS), Araras, São Paulo, Brazil.

### Statistical analysis

In the captured ants, we calculated the absolute frequency, accumulated absolute frequency, relative frequency, and cumulative relative frequency of the isolated microorganisms. Fisher’s exact test was used to evaluate the hypothesis that the proportions of microorganisms were related to the type of ant. The confidence interval for differences in the evaluated proportions was used [15–17]. Statistical analyses were performed in the Department of Statistics, Federal University of Goiás, Goiás, Brazil.

## Results

Seventy ants were captured from the evaluated sites; of which, 62.8% were of the genus *Paratrechina* and 25.7% were *Monomorium floricola*. Among the other ants found 7.1% were from the genus *Dorymyrmex*, 2.8% from *Pheidole* and 1.4% from *Brachymyrmex* (Table 1).

**Table 1:** **Frequencies of ants and microorganisms in a hospital in São Paulo county, March 2012 to February 2013**

Ant species	Absolute frequency of ants	Absolute frequency of microorganisms
*Paratrechina* spp*.*	44	24 *Bacillus* spp.; 7 *Listeria* spp.; 2 *Arcanobacterium* spp.; 1 *Streptococcus* spp.; 1 *Pseudomonas aeruginosa*; 1 *Micrococcus luteus*; 1 *Sthaphylococcus epidermidis*
*M. floricola*	18	4 *Bacillus* spp.; 4 *Streptococcus* spp.; 3 *Pseudomonas aeruginosa*; 3 *Staphylococcus aureus;* 2 *Proteus* spp.; 2 no microbial growth
*Dorymyrmex* spp.	5	3 *Klebsiella* spp.*;* 2 *Bacillus* spp.
*Pheidole* spp*.*	2	2 *Bacillus* spp.
*Brachymyrmex* spp*.*	1	1 *Pseudomonas aeruginosa*

Among the bacteria isolated from the integument of captured ants, 45.7% were *Bacillus* spp., and other bacteria were *Listeria* spp. (10%), *Streptococcus* spp. (7.1%), *Pseudomonas aeruginosa* (7.1%), *Klebsiella* spp. (4.2%), *Staphylococcus aureus* (4.2%), *Arcanobacterium* spp. (2.8%), *Proteus* spp. (2.8%), *Micrococcus luteus* (1.4%) and *Staphylococcus epidermidis* (1.4%). In addition, 12.8% of the ants showed no microbial growth. Among the isolated bacteria, ~68.8% were Gram-positive bacilli, 16.4% were Gram-positive cocci and about 14.7% were Gram-negative bacilli. Regarding Gram-negative bacilli, 55.5% were isolated from *M. floricola* ants, 22.2% from *Dorymyrmex* spp., 11.1% from *Brachymyrmex* spp. and 11.1% from *Pheidole* spp. For the Gram-positive bacilli, 78.5% were isolated from *Paratrechina* spp., 9.5% from *M. floricola*, 7.15% from *Dorymyrmex* spp. and 4.7% from *Pheidole* spp. Gram-positive cocci were isolated from two different types of ants, 70% from the integument of *M. floricola* and 30% from *Paratrechina* spp*.* (Table 2).

**Table 2:** **Bacteria isolated from ants captured in a hospital in São Paulo county, March 2012 to February 2013**

Microorganisms			
Ants	Gram-negative bacilli	Gram-positive bacilli	Gram-positive cocci
*Brachymyrmex* spp.	1	0	0
*Dorymyrmex* spp.	2	3	0
*M. floricola*	5	4	7
*Paratrechina* spp.	1	33	3
*Pheidole* spp.	0	2	0

Considering only *M. floricola* and *Paratrechina* spp., the Fisher’s exact test was used to evaluate the hypothesis that the proportions of organisms were related to the type of ants (Table 3). Because there was a relationship between the variables, we tried to measure individual differences in the proportions of bacteria isolated from *M. floricola* and *Paratrechina* spp. These individual differences were evaluated using confidence intervals for differences in proportions. Table 4 shows a summary of the proportions, confidence intervals and the conclusions of these intervals.

**Table 3:** **Relationship between ants and bacteria from a hospital in São Paulo county, March 2012 to February 2013**

Ants	Arc	Bac	Str	Lis	Mic	Pro	Pse	NMG	Sta	Ste
*M. floricola*	0	4	4	0	0	2	3	2	3	0
*Paratrechina* spp.	2	24	1	7	1	0	1	7	0	1

Fisher’s exact test: *p* value = 9.93e-05.

Arc: *Arcanobacterium* spp.; Bac: *Bacillus* spp.; Est: *Streptococcus* spp.; Lis: *Listeria* spp*.*; Mic: *M. luteus*; Pro: *Proteus* spp.; Pse: *P. aeruginosa*; NMG: No Microbial Growth; Sta: *S. aureus*; Ste: *S. epidermidis.*

**Table 4:** **Proportions of bacteria in ants in a hospital in São Paulo county, March 2012 to February 2013**

	***Arcanobacterium***spp***.***	***Bacillus***spp.	***Streptococcus***spp.	***Listeria***spp.	***M. luteus***
*M. floricola*	0.00%	22.22%	22.22%	0.00%	0.00%
*Paratrechina* spp*.*	4.55%	54.55%	2.27%	15.91%	2.27%
95% CI	(−15.26%; 13.54%)	(−52.92%; −4.99%)	(−15.26%; 13.54%)	(−29.49%; 2.76%)	(−11.93%; 15.68%)
Result	NS*	*Paratrechina* spp. *< M. floricola*	*Paratrechina* spp. *< M. floricola*	NS*	NS*
	***Proteus*** **spp.**	***P. aeruginosa***	**Without growth**	***S. aureus***	***S. epidermidis***
*M. floricola*	11.11%	16.67%	11.11%	16.67%	0.00%
*Paratrechina* spp*.*	0.00%	2.27%	15.91%	0.00%	2.27%
95% CI	(2.32%; 33.01%)	(0.80%; 37.43%)	(−21.35%; 18.76%)	(5.79%; 39.43%)	(−11.93%; 15.68%)
Result	*Paratrechina* spp*. < M. floricola*	*Paratrechina* spp. *< M. floricola*	NS*	*Paratrechina* spp*. < M. floricola*	NS*

*NS means: not statistically significant.

We observed that the presence of *Streptococcus* spp, *Proteus* spp, *P. aeruginosa* and *S. aureus* was greater in *M. florícola* than *Paratrechina* spp. In contrast, *Bacillus* spp. were more prevalent in *Paratrechina* spp. compared with *M. floricola*. These differences were significant, with 95% confidence.

The environment with the highest percentage of ants captured was the medical clinic, with 80% of the total, followed by outdoor areas (8.5%), obstetric unit (8.5%) and reception room (2.8%). No ants were found in the kitchen, surgical centres, paediatric clinic and ICU. These data are listed in Table 5. Considering only the environments where ants were captured, in the medical clinic, 64.3% of the ants were from the genus *Paratrechina*, followed by *M. floricola* (26.8%), *Pheidole* (3.6%), *Dorymyrmex* spp. (3.6%) and *Brachymyrmex* spp. (1.8%). Outside the hospital, 50% of the ants are were *Dorymyrmex* spp., while the other 50% were *Paratrechina* spp. In the obstetrics unit, 83.3% of the ants were *Pratrechina* spp., followed by 16.7% *M. floricola*. In the reception area, all the ants were *M. floricola.*

**Table 5:** **Number of ants captured according to hospital environment, March 2012 to February 2013**

Environment	Total ants	*Days with observation
Medical clinic	56	6
External area	6	3
Obstetric clinic	6	2
Reception room	2	1
Kitchen	0	0
Surgical centre	0	0
Paediatric clinic	0	0
Intensive care unit	0	0

*Days with observation of ants within the specified environment.

## Discussion

Considering only the places where ants were captured, in the medical clinic, 64.3% of the ants found were from the genus *Paratrechina*. Fowler *et al.* [9], Bueno *et al.* [10] and Zarzuela *et al.* [11] also recorded this genus in Brazilian hospitals. *Monomorium floricola* species represented 26.8% of the ants found in the same environment. This species is considered to be one of the major existing exotic species of ant in Bueno and Campos-Farinha, Brazil [4]. The genus *Pheidole* represented 3.6% of ants captured in the same environment, and this is a native species in Brazil and considered by Campos-Farinha *et al.* [18] to be a major urban plague. The genus *Dorymyrmex* accounted for 3.6% of ants captured in the clinic, while *Brachymyrmex* spp. represented 1.8%. In households, ants use cracks in tiles, windows and doorframes to form nests, and the construction of nests is not prevented by well-maintained physical structures [19].

From the 70 samples of aseptically collected ants, 61 (87.1%) had pathogenic bacteria in their integuments, which agrees with previous studies [1–3, 5, 7–9, 12, 20–23]. Regarding Gram-positive bacilli, it is noteworthy that their ability to form endospores allows them to become established in places of great environmental instability. *Streptococcus* spp. and *Klebsiella* spp. have been isolated from ants in hospital environments and identified as multiresistant to antibiotics [20]. *Pseudomonas aeruginosa* represented 7.1% of bacteria found. It is frequently isolated from clinical samples, causing infections that are usually associated with sites that have a tendency towards humidity, such as tracheostomy, indwelling catheters, burns and exudative wounds.

According to Tresoldi *et al.* [24], the frequency of microorganisms isolated from nosocomial infection was 56.5% Gram-negative bacilli, 20.9% Gram-positive cocci and 9% yeast. The possibility of transmission of microorganisms and their presence in the hospital environment may act to maintain or increase such findings.

In our study, among the bacteria isolated from the integument of captured ants, 4.2% were *Klebsiella* spp. Cassettari *et al.* [25] reported that this bacterium was responsible for a 5.6% rate of urinary tract infections, conjunctivitis and bacteraemia in a public hospital in São Paulo, Brazil. They also showed that *S. aureus* accounted for 4.2% of the bacteria found, and according to Nagao *et al.* [26], it is an important causative agent of nosocomial infection, mainly primary bloodstream infections. Infections caused by *S. epidermidis* include endocarditis, intravenous catheter infection, peritonitis associated with peritoneal dialysis catheter, bacteraemia, wound infections, prosthesis infections and infections of the upper airways [7, 14].

In studies by Costa *et al.* [7], bacteria were isolated from ants in hospitals; particularly *Pseudomonas* spp., *Staphylococcus* spp. and *Micrococcus* spp. Carneiro *et al.* [27] have also observed *Staphylococcus* spp. and *Klebsiella* spp. as pathogenic bacteria carried by ants.

Some of the bacteria isolated in the present study may present a risk to health, so it is necessary to consider several factors that may cause infection. Considering that ants can be one of the factors responsible for nosocomial infection, preventive measures and control of urban ants should be implemented to reduce the risks that these vectors present.

## Conclusions

Our results are clearly of relevance to public health, particularly hospital infection control. We believe that knowledge about the biology, ecology and habits of ants may help to reduce the problems caused by ants in hospitals. The presence of ants in this environment must be seen as a warning to the Commission of Hospital Infection Control and all multidisciplinary hospital staff. Therefore, the development of basic preventive measures and the control of ants must be undertaken.
